# A community survey of coverage and adverse events following country-wide triple-drug mass drug administration for lymphatic filariasis elimination, Samoa 2018

**DOI:** 10.1371/journal.pntd.0008854

**Published:** 2020-11-30

**Authors:** Gabriela A. Willis, Helen J. Mayfield, Therese Kearns, Take Naseri, Robert Thomsen, Katherine Gass, Sarah Sheridan, Patricia M. Graves, Colleen L. Lau

**Affiliations:** 1 Research School of Population Health, Australian National University, Canberra, Australia; 2 Menzies School of Health Research, Charles Darwin University, Brisbane, Australia; 3 Ministry of Health, Apia, Samoa; 4 Neglected Tropical Diseases Support Center, The Task Force for Global Heath, Atlanta, Georgia, United States of America; 5 School of Public Health and Community Medicine, University of New South Wales, Sydney, Australia; 6 College of Public Health, Medical and Veterinary Sciences, James Cook University, Cairns, Australia; University Hospital Bonn, GERMANY

## Abstract

The Global Programme to Eliminate Lymphatic Filariasis has made considerable progress but is experiencing challenges in meeting targets in some countries. Recent World Health Organization guidelines have recommended two rounds of triple-drug therapy with ivermectin, diethylcarbamazine (DEC), and albendazole (IDA), in areas where mass drug administration (MDA) results with two drugs (DEC and albendazole) have been suboptimal, as is the case in Samoa. In August 2018, Samoa was the first country in the world to implement countrywide triple-drug MDA. This paper aims to describe Samoa’s experience with program coverage and adverse events (AEs) in the first round of triple-drug MDA. We conducted a large cross-sectional community survey to assess MDA awareness, reach, compliance, coverage and AEs in September/October 2018, 7–11 weeks after the first round of triple-drug MDA. In our sample of 4420 people aged ≥2 years (2.2% of the population), age-adjusted estimates indicated that 89.0% of the eligible population were offered MDA, 83.9% of the eligible population took MDA (program coverage), and 80.2% of the total population took MDA (epidemiological coverage). Overall, 83.8% (2986/3563) reported that they did not feel unwell at all after taking MDA. Mild AEs (feeling unwell but able to do normal everyday things) were reported by 13.3% (476/3563) and moderate or severe AEs (feeling unwell and being unable to do normal everyday activities such as going to work or school) by 2.9% (103/3563) of participants. This study following the 2018 triple-drug MDA in Samoa demonstrated a high reported program awareness and reach of 90.8% and 89.0%, respectively. Age-adjusted program coverage of 83.9% of the total population showed that MDA was well accepted and well tolerated by the community.

## Author summary

Lymphatic filariasis is a disease caused by worms and transmitted between humans by mosquitoes. A global program underway aims to eliminate lymphatic filariasis as a public health problem by distributing two deworming drugs to the whole population once a year for at least five years. In some countries, including Samoa, this strategy has not been sufficient to eliminate transmission. A new drug has been added, and in 2018, Samoa was the first country in the world to distribute country-wide triple-drug mass drug administration (MDA) using ivermectin, diethylcarbamazine, and albendazole. This study reports on the coverage achieved (percentage of people who reported taking the drugs) and adverse events after taking the drugs. The study was a large community survey of over 4000 people, done 7–11 weeks after the distribution of the first round of triple-drug MDA. We found that the program reached and offered MDA to approximately 90% of the whole population, and approximately 80% of the whole population swallowed the drugs. We also collected data on whether people felt unwell after taking the pills, and found that 84% of people taking the pills did not feel unwell at all, while 13% reported feeling unwell afterwards but were able to do normal everyday activities, and 3% reported that they had felt unwell and that it stopped them doing normal everyday activities, such as going to school or work.

## Introduction

Lymphatic filariasis (LF) is a disabling and disfiguring neglected tropical disease caused by infection with three species of filarial worms (*Wuchereria bancrofti*, *Brugia malayi*, and *B*. *timori*) [1]. Transmission is by mosquito vectors, which deposit larvae onto the skin when biting humans. The larvae enter the body and migrate to the lymphatic system where they develop into adult worms. Microfilariae (immature larvae), produced by the adult worms, circulate in the blood and infect biting mosquitos, thus enabling ongoing transmission [2]. Chronic manifestations include lymphoedema, typically in the lower limbs, elephantiasis (skin/tissue thickening), and scrotal hydrocoele, which can cause significant disability and social stigma [1,3]. Laboratory diagnostic tests include detection of microfilariae and circulating filarial antigen (Ag) in the blood [4].

In 2014, it was estimated that there were almost 68 million persons living with LF globally, including 36 million microfilaria carriers, 19 million hydrocoele cases, and 17 million lymphoedema cases [5]. LF has been identified by the International Task Force for Disease Eradication as ‘eradicable’ or ‘potentially eradicable’ [6]. The World Health Organization (WHO) launched the Global Programme to Eliminate Lymphatic Filariasis (GPELF) in 2000 with the aim of eliminating LF as a public health problem through two approaches. The first component consists of community-wide ‘mass drug administration’ (MDA) delivered annually over 4–6 years, to reduce microfilariae prevalence in a population to the point that transmission is considered unsustainable. A second component of the program provides care to people already affected by chronic complications such as lymphoedema and hydrocoele [7,8]. Although significant progress has been made, with an estimated 97 million cases of LF being prevented or cured by 2013 [5], the program faces challenges that have slowed progress towards elimination in some countries. By 2018, 7.7 billion treatments had been delivered to >910 million people in 68 LF-endemic countries, and 14 countries had officially achieved elimination status, but 893 million people in 49 LF endemic countries still required MDA [8].

LF is endemic to Samoa, an island country in the South Pacific. Despite delivering 10 rounds of MDA prior to 1999, eight rounds under the Pacific Programme to Eliminate Lymphatic Filariasis (PacELF) (1999–2003, 2006, 2008, and 2011) and two additional rounds in one region of the country (Northwest Upolu 2015 and 2017) [9], a Transmission Assessment Survey (TAS) in 2017 showed evidence of ongoing transmission. According to recently published guidelines, in settings where onchocerciasis is not endemic and where effectiveness of MDA has been suboptimal, as is the case in Samoa, WHO recommends the use of two annual rounds of a triple drug combination (ivermectin, diethylcarbamazine, albendazole [IDA]), a regime shown to be potentially more effective for achieving sustained clearance of microfilariae [10,11].

In August 2018, Samoa was the first in the world to implement country-wide triple drug MDA [9]. In preparation for MDA, Samoa developed a National Action Plan for Elimination of LF, with the following objectives: i) to stop transmission of LF and prevent new infections; ii) to ensure the provision of basic care for people living with disability due to LF; and iii) to enhance post-MDA surveillance towards validation of elimination by 2024. Samoa also established a National LF Control and Elimination Taskforce to oversee preparation, implementation, monitoring and evaluation of the National Action Plan. The 2018 Samoan campaign aimed to deliver MDA to all eligible individuals through primary and secondary schools, house-to-house visits, workplaces, churches, booths, and central distribution points within communities and ports. Community awareness and advocacy campaigns were conducted through and with schools, workplaces, institutions, churches, and villages. To enhance acceptability of MDA, consultations were conducted to seek engagement and support from multiple stakeholders, including national and local policy-makers, community leaders, religious leaders, school principals, doctors, and ministerial staff [12].

In Samoa, the first round of triple-drug MDA was implemented by the Ministry of Health over two weeks in August 2018 by a team of 1600 community drug distributors. A single oral treatment of IDA was given; the number of tablets was calculated based on body weight (ivermectin 150–200μg/kg, diethylcarbamazine [DEC] 6mg/kg, and albendazole 400mg) to determine recommended doses in eight weight categories, and simplified dose charts were used by drug distributors (S1 Table). Directly observed treatment was used whenever possible, and fingernails were marked with indelible ink to indicate participation. MDA was not offered to pregnant women, children aged <2 years, elderly aged >80 years (unless they wished to take the medications), the severely ill, lactating mothers in the first seven days after birth, epileptic children who had experienced a seizure in the previous three weeks, people with heart problems who were experiencing shortness of breath, and people with allergies to any worm medications. Children aged 2–4 years were offered DEC and albendazole (DA), while children aged ≥5 years and >15 kg were given IDA. Therefore, children aged 2–4 years received two tablets (one DEC and one albendazole), while those aged ≥5 years received between three and 17 tablets, depending on weight.

MDA coverage in the past has usually been reported as ‘program coverage’, based on summaries of numbers of pills distributed and persons treated from distribution records [13]. There have been few population representative surveys of MDA coverage in the Pacific region. In neighbouring American Samoa, coverage for the 2002 MDA round was estimated to be 54.3% from interviews with 153 participants in a community cluster survey (one person per household, 12 households per village in 20 villages), which was similar to the reported program coverage (49%) [14]. Following the 2004 MDA round in American Samoa, a simple random sample of 1597 persons living in 278 households found a coverage of 81.6%, in comparison to a program coverage estimate of 65% [14]. Achieving high levels of coverage over one or more rounds of MDA is critical to achieving elimination of LF, and taking MDA (also in American Samoa) was significantly associated with a reduction in Ag positivity [15].

The Samoa Ministry of Health (MOH) assessed reach, compliance, and coverage using the WHO recommended Supervisor’s Coverage Tool (SCT), in which a single individual is surveyed from each of 20 randomly selected houses in a supervisory area (such as a village or other administrative unit) [16]. The SCT was conducted in three villages (Faleasiu, Leauva’a, and Nofoali’i) within two weeks post-MDA, and coverage reported was 90% or higher in all three villages (S2 Table). However, the SCT is intended to be a rapid in-process monitoring tool and does not give a representative estimate of population coverage.

Mild to moderate systemic adverse events (AEs) are common following MDA, including fever, headache, dizziness, malaise, myalgia, fatigue and gastrointestinal upset. Localised AEs, thought to arise from the death of adult filarial worms in lymphatic vessels, including subcutaneous or scrotal nodules, spermatic cord swelling, lymphadenitis, or new onset hydrocoele or lymphoedema, occur less frequently [17]. The Samoa MOH developed a system for reporting, managing, and investigating adverse events that could potentially be related to the 2018 MDA. Community drug distributors were provided with training and information to answer common questions from community members, with designated doctors being on call to assess and investigate any severe AEs and manage risk communication. According to the MOH, a total of 65 people presented to a public health facility with MDA-related AEs (S3 Table); 28 (43.1%) were aged 2–10 years and the most commonly reported symptoms were dizziness, nauseas, lethargy, and a rash (S4 Table). No serious AEs were reported that were assessed as related to MDA. During the MDA distribution period, four deaths were reported in persons who took the medications, but immediate investigation by MOH-designated medical officers determined that the causes of death were unrelated to the MDA medications. Information regarding these deaths have been reported to the WHO, but details have not been included here to protect the confidentiality of individuals.

The Surveillance and Monitoring to Eliminate Lymphatic Filariasis and Scabies from Samoa (SaMELFS Samoa 2018) study was a cross-sectional community survey conducted 7–11 weeks after the first round of triple-drug MDA in 2018. While the MDA, SCT, and the official system for reporting and investigating adverse events were implemented by the Ministry of Health, the SaMELFS study was well-placed to provide a large population-representative assessment of adverse events related to the first country-wide use of triple-drug MDA. This paper reports findings from the SaMELFS community survey regarding MDA program awareness, reach, coverage, compliance, and self-reported adverse events.

## Methods

### Ethics statement

All field activities were carried out in a culturally appropriate and sensitive manner with bilingual local field teams, who received training prior to the study. Verbal approval to conduct the study in each village was sought from community leaders, including the village chief, mayor and/or church leaders. Community leaders disseminated information about the study prior to the visits and assisted with organizing the convenience survey. Prior to enrolment, participants were given verbal information about the study (plus written information if appropriate) in Samoan or English, and written informed consent was obtained from each participant or parent/guardian for minors aged <18 years. For the convenience survey, children were eligible if they had a written consent form from a parent/guardian and were accompanied by a parent or another person (e.g. older sibling or other relative) aged ≥15 years. Verbal assent was obtained from minors in addition to written informed consent from a parent/guardian. Ethical approval was obtained from human research ethics committees at the Samoa Ministry of Health and The Australian National University (protocol 2018/341). The study was conducted in collaboration with the Samoa Department of Health, WHO Samoa country office, Samoa Red Cross, The Task Force for Global Health, and the United States Centers for Disease Control and Prevention.

### Study location

Samoa (previously known as Western Samoa) is an independent country in the South Pacific (latitude 13° 35 South, longitude 172° 20 West) with a population of ~199,000 [18]. Over 90% of the population live on two main islands: Upolu and Savai’i. Samoa is divided into four administrative regions: Apia Urban Area (AUA), Northwest Upolu (NWU), Rest of Upolu (ROU), and Savai’i (SAV). There are ~338 villages, with average population size of ~580 (range <20 to 4300) [19]. The majority of the population reside on the main island of Upolu, split between the mostly urban AUA (~37,500 residents), and the mainly rural NWU (~69,300 residents) and ROU (~45,600 residents–including on the several smaller islands). Savai’i (SAV) has approximately 43,500 residents and is predominantly rural.

### Study design

The SaMELFS Samoa 2018 study was conducted with the primary aims of assessing baseline LF prevalence in Samoa before the first round of triple drug MDA, and to identify ‘hotspots’ of transmission with high Ag prevalence (results to be reported in another publication). It included a population representative community-based cross-sectional cluster survey, which was delayed due to logistic reasons and took place in September/October 2018, 7–11 weeks post-MDA, instead of prior to MDA as intended. Consequently, the SaMELFS 2018 survey was ideally placed to provide data on MDA reach, compliance, coverage, and self-reported adverse events.

### Participant sampling and recruitment

**Fig 1:**
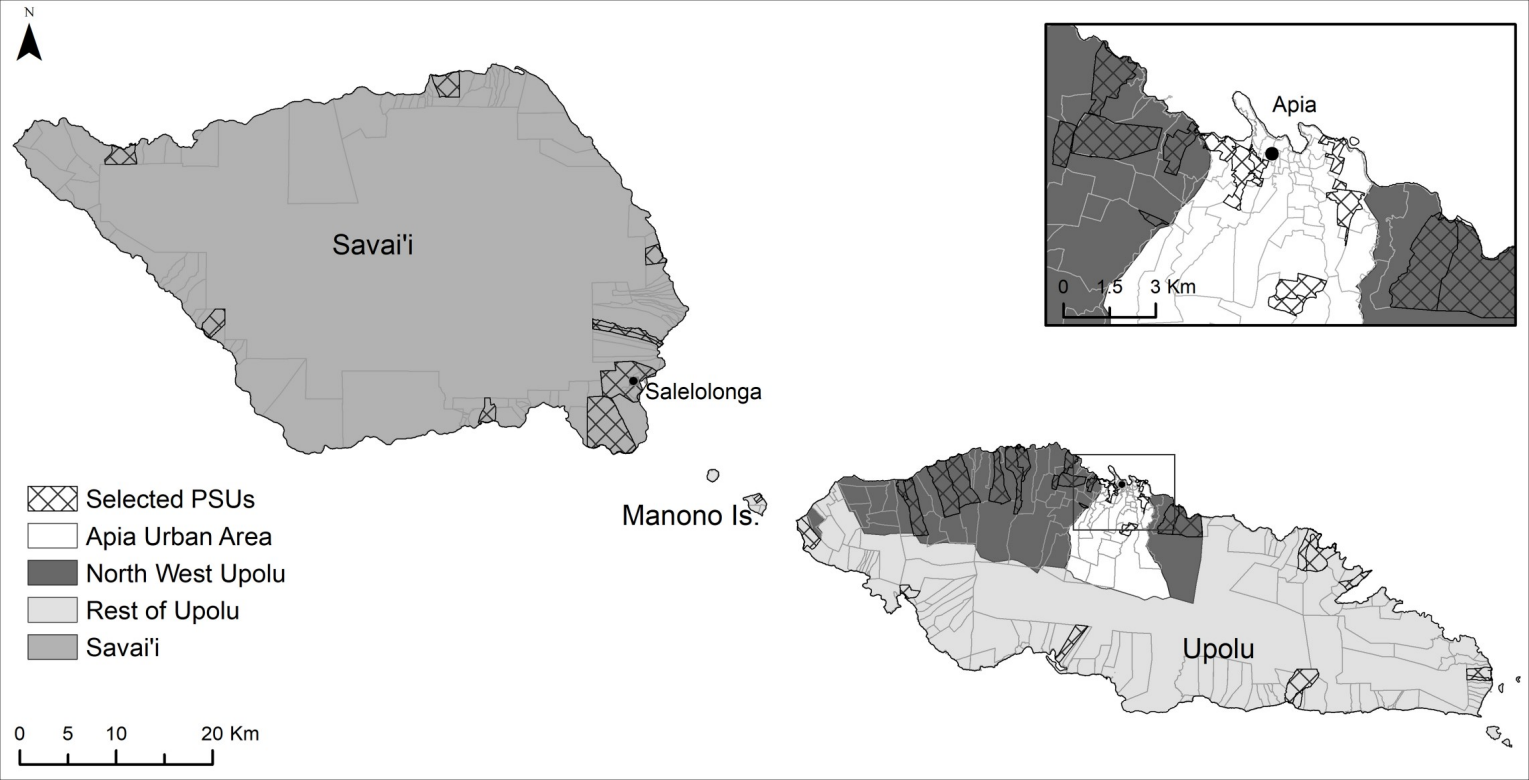
Map of Samoa with administrative region boundaries and selected villages.

Participants were sampled from 35 primary sampling units (PSUs) located throughout Upolu, Savai’i, and Manono Islands (Fig 1). Five PSUs were purposively sampled (three in NWU, one in ROU, and one in SAV) in consultation with the Samoa MOH, as they were suspected to be transmission ‘hotspots’ based on local knowledge and results of previous surveys. The remaining 30 PSUs were randomly selected using a line list of villages from the 2016 census. Of the 30 randomly selected villages, eight were very small (total population <600) and an adjacent village was added to ensure that target sample size for the PSU was achievable. Therefore, the 35 PSUs included a total of 43 individual villages.

The target sample size was 4400, comprising 2000 children aged 5–9 years, 2000 people aged ≥10 years, and 400 children aged <5 years. Sample size calculations were based on numbers required to detect a critical threshold of 2% Ag prevalence in each age group, with a 5% chance of type 1 error, 75% power (when true prevalence is 1%), and a design effect of 2.0. This equated to a target of 57 children aged 5–9 years and 57 adults aged ≥10 years in each PSU. Children aged 2–4 years were not tested for Ag, but all children (of any age) in selected households were invited to participate in a linked scabies study.

In each PSU, we sampled participants in the selected communities via one of two sampling strategies: i) Household survey (all ages); and ii) Convenience survey (children aged 5–9 years). The convenience survey component was designed to ensure recruitment of the target number of children aged 5–9 years old. These components will be described in further detail below.

#### Household survey

We selected 15 households in each PSU using detailed village aerial maps obtained from Google Maps. Firstly, all buildings resembling a house were numbered sequentially through a “virtual walk”. A house was then randomly chosen as the starting point for household selection, and the remaining 14 houses were selected at equal intervals from this starting point based on the order in which they were numbered. If a selected household was uninhabited, we replaced it with the nearest inhabited household. If nobody was home at the time of the visit, the house was revisited later in the day and/or when revisiting the village on another day. If household members were still absent at the time of the second attempted visit, we replaced the selected household with the nearest inhabited household.

All household members aged ≥2 years were invited to participate in the LF study (children aged <2 years were only invited to participate in a linked scabies survey). An individual was considered a ‘household member’ if the house was their primary place of residence, and/or if they slept there the previous night. If eligible household members were not present but were expected to return later in the day, we arranged to revisit the house to include them.

#### Convenience survey

All children aged 5–9 years who had not been sampled in the household survey were invited with a parent/guardian to a central place in the village e.g. a school, community hall, church or large *fale* (traditional Samoan open house) for participation in the convenience survey. The number of children enrolled via the convenience survey was dependent on the number of children aged 5–9 years who had already been enrolled via the household survey, with enrolment stopping once the target of 57 had been reached. If insufficient numbers of children attended, we liaised with community leaders to invite more participants.

### Data collection

All participants or parent/guardians (for minors aged <18 years) completed a questionnaire and participants aged ≥5 years had a blood specimen collected and tested for LF Ag and antibodies.

#### Questionnaires

Interviewers obtained informed consent, enrolled participants and completed questionnaires verbally in Samoan or English, depending on the participant’s preference. Parents/guardians answered questions for minors. Interviewers entered data directly into an electronic record on smartphones using Secure Data Kit software (SDK, Atlanta, GA). For participants aged ≥5 years we collected data on demographics, information on MDA participation in 2018, reasons for not participating, whether they felt unwell after taking the pills, whether they were able to do normal everyday activities if unwell, presence of indelible ink marks on their finger from MDA participation, and participation in MDA rounds prior to 2018. For participants aged 2–4 years, we collected simplified data on demographics and MDA participation in 2018.

#### Specimen collection and testing

For each participant aged ≥5 years, we collected up to 400μl of blood by finger prick into a heparin microtainer. Samples were stored in a portable cooler until the team returned to the field laboratory, where they were refrigerated and tested the following day (or on Monday if collected on a Saturday). Blood samples were tested for circulating filarial Ag for *W*.*bancrofti* using the Filariasis Test Strip (FTS; Alere, Scarborough, ME). Positive tests were followed by confirmatory repeat FTS if sufficient blood was available.

### Data management

We collected enrolment and questionnaire data electronically on smartphones and uploaded regularly to a cloud-based electronic database using SDK, and data were stored on a SQL secure server. Each participant was assigned a unique scannable QR code to link their enrolment/consent form, questionnaire, blood specimen, and laboratory results.

### Data analyses

Mild AE was defined as feeling unwell after taking MDA but still able to take do normal everyday activities. Those who reported feeling unwell after taking MDA and being unable to do normal everyday activities (e.g. missed school or work) were classified together as moderate/severe AEs. Our study was a community survey rather than a clinical trial, and was not designed to differentiate between moderate and severe events because i) the survey was conducted 7–11 weeks post-MDA, and potentially subject to recall bias; ii) most of our field team were not clinically trained, so it was not possible to accurately classify AEs into moderate or severe, or to assess likelihood of casual links between AEs and MDA; iii) the official reporting, management and investigation of adverse events during and immediately after MDA was conducted by the Ministry of Health (as described above), and these activities were not part of the SaMELFS study.

We analysed data using Stata/IC (StataCorp LLC, Texas USA, Version 15.0). A p-value of <0.05 was considered statistically significant. We performed descriptive analyses to estimate reported MDA program awareness, reach, coverage, and compliance, as well as reported adverse events. S1 Fig and S5 Table show the flowchart and formulae for deriving each of the metrics. We used Chi-squared tests to compare proportions between population sub-groups and Clopper-Pearson binomial exact methods to estimate 95% confidence intervals (CI). Pearson correlation coefficient was used to measure linear correlation between variables. We used 2011 and 2016 Samoa Bureau of Statistics census data to make demographic comparisons with the general population [20]. Prevalence estimates were standardized for age using the ‘stdize’ option in the ‘proportion’ command in Stata/IC, with ‘stdweights’ as the proportion of the population in each age group (categorized into five-year intervals).

Clustering of coverage was examined using multilevel hierarchical modelling that allowed for correlation of observations by region (n = 4), PSU (n = 35), and households (n = 499) as random effects (Stata command *melogit*). Children from the convenience survey were not included in the models because household-level data were not available. Age and gender were included in the models as fixed effects. Intraclass correlation coefficients (ICCs) with corresponding 95% confidence intervals were obtained from the multivariable models.

### Spatial data and mapping

Spatial data on country, island, region, and village boundaries in Samoa were obtained from the Pacific Data Hub (pacificdata.org) and DIVA-GIS (diva-gis.org). Geographic information systems (GIS) software (ArcGIS v10.4.1, Environmental Systems Research Institute, Redlands CA) was used to manage spatial data and produce maps.

## Results

### Study population and antigen prevalence

We recruited a total of 4420 participants from 35 PSUs (43 villages) (~2.2% of the total population), including 281 children aged 2–4 years (6.4%), 1942 children aged 5–9 years (43.9%), and 1999 aged ≥10 years (45.2%) (Table 1). A total of 4222 participants were aged ≥2 years and eligible for the LF survey. Of the 4222 participants, questionnaire data were available for 4213 (99.8%), including 280 children aged 2–4 years, 1940 aged 5–9 years, and 1993 aged ≥10 years.

**Table 1:** Summary of study population demographic characteristics.

	Household survey (N = 2878)	Convenience survey (N = 1542)	All participants (N = 4420)
**Age groups** (years) n (%)			
0–1	198 (6.9)	N/A	198 (4.5)
2–4	281 (9.8)	N/A	281 (6.4)
5–9	400 (13.9)	1542 (100)	1942 (43.9)
≥ 10	1999 (69.5)	N/A	1999 (45.2)
**Age** (years)			
Range	0–90	5–9	0–90
Mean ± SD	25.3 ± 20.9	7.1 ± 1.4	18.9 ± 19.2
**Sex** n (%)			
Male	1361 (47.3)	814 (52.8)	2175 (49.2)
Female	1517 (52.7)	728 (47.2)	2245 (50.8)
**Region** n (%)			
AUA	492 (17.1)	249 (16.1)	741 (16.8)
NWU	1182 (41.1)	645 (41.8)	1827 (41.3)
ROU	667 (23.2)	323 (20.9)	990 (22.4)
SAV	537 (18.7)	325 (21.1)	862 (19.5)

Of the 209 participants who were not eligible for MDA, 198 were too young (aged <2 years), seven were pregnant, three were ill, and one did not provide a reason. A total of 2680 participants aged ≥2 years (63.5%) were sampled via the randomly selected households, and 1542 (36.5%) participants were sampled via the convenience survey. Greater than 90% of households approached agreed to participate. We included a total of 499 households, and an average of 14.3 households per PSU, representing 6.2% of the total estimated 8006 households in sampled villages. Median household size was six people (range 1–20).

**Fig 2:**
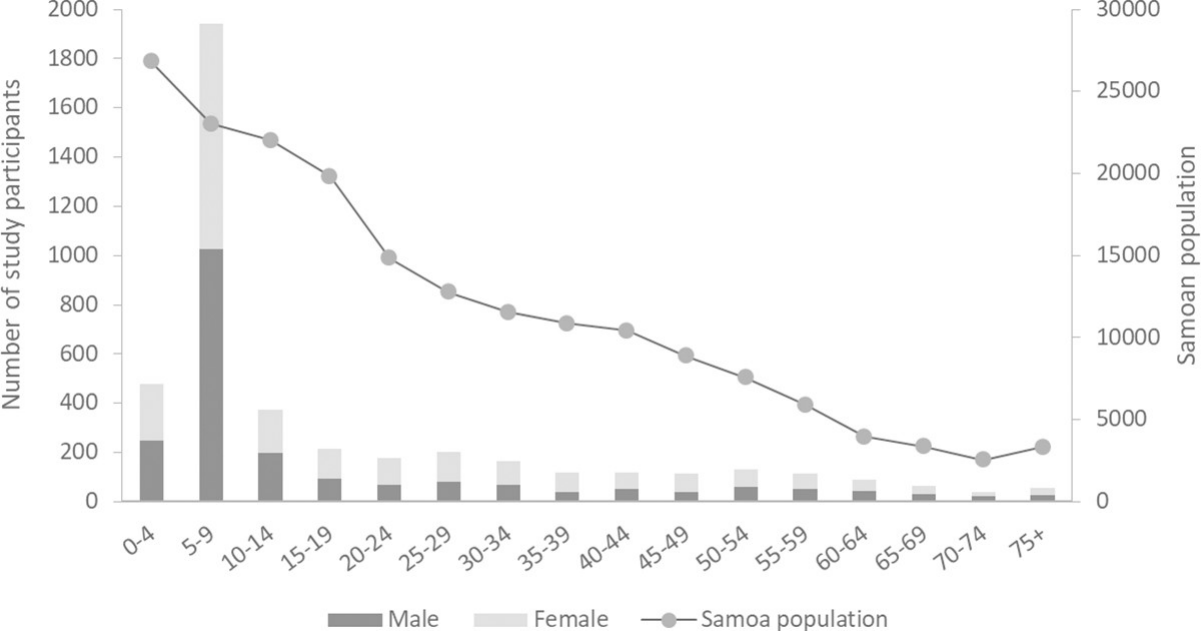
Age distribution of study participants (primary axis) and Samoan population (2011 census) (secondary axis).

Age distribution relative to the Samoan population was skewed, with overrepresentation of children aged 5–9 years due to the recruitment strategy and primary study aim of the LF Ag seroprevalence study (Fig 2). There was approximately equal sex distribution, with 50.8% of participants being female. Overall, 1827 (41.3%) were sampled from NWU, with 990 (22.4%) from ROU, 741 (16.8%) from AUA, and 862 (19.5%) from SAV. This was broadly representative of the regional population distribution (35.3% NWU, 23.3% ROU, 19.1% AUA, 22.2% SAV) [19].

Ag prevalence was 1.5% (95% CI 1.0–2.1%) in participants aged 5–9 years (28 positives out of 1923 valid results), and 4.9% (95% CI 4.0–5.9%) in those aged ≥10 years (94 positives out of 1929 valid results). Age-adjusted Ag prevalence was 4.3% (95% CI 3.5–5.2%) for all regions combined, 3.5% (95% CI 2.1–5.7%) for AUA, 6.2% (95% CI 4.9–7.9%) for NWU, 1.8% (95% CI 1.0–3.4%) for ROU, and 3.3% (95% CI 2.0–5.6%) for SAV.

### MDA awareness, reach, and coverage

**Fig 3:**
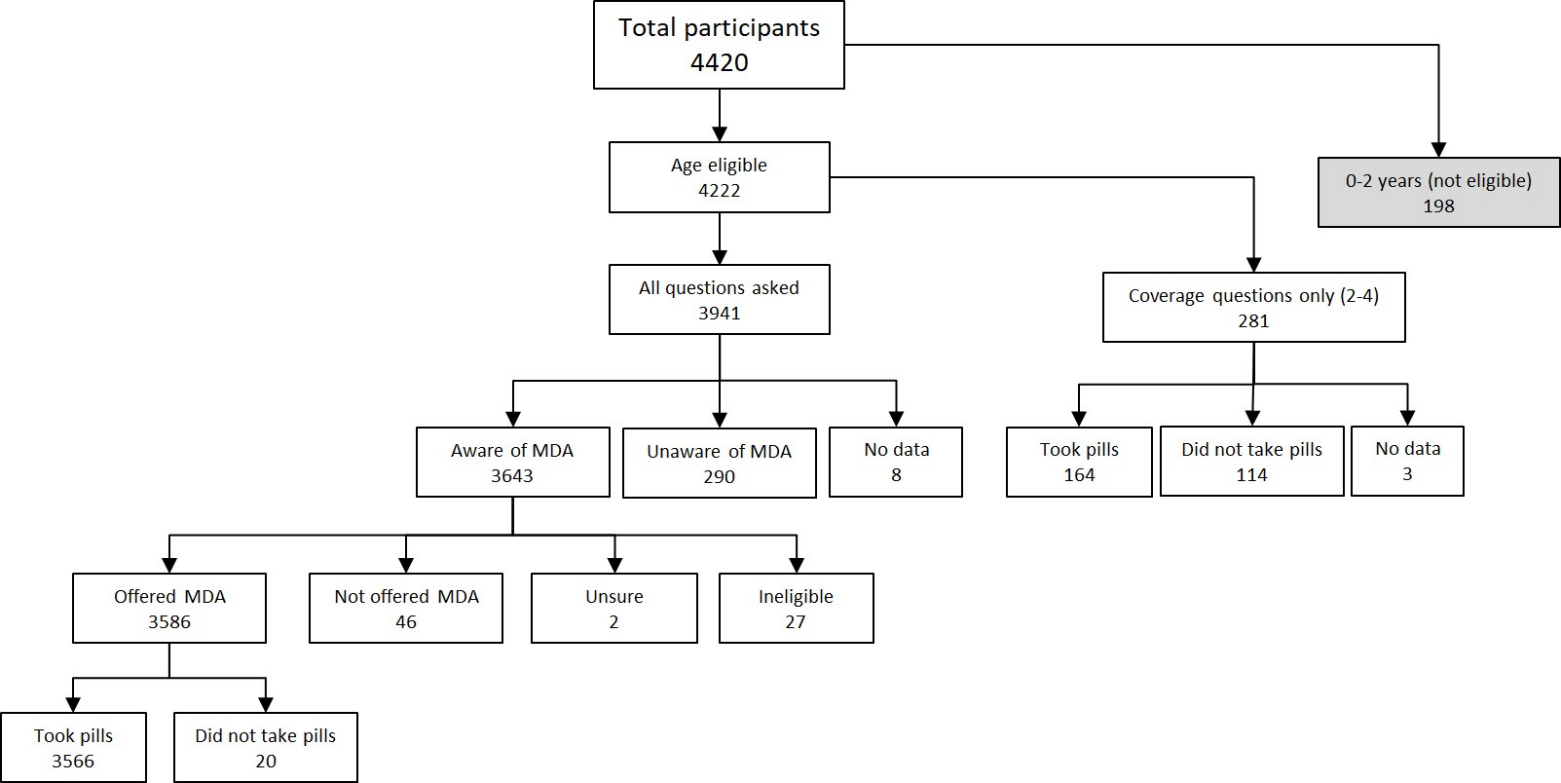
Study participant flowchart.

In participants aged ≥5 years where questionnaire data were available, 92.6% (3643/3933) of participants or their parent/guardian reported being aware of the MDA (Fig 3 and Table 2). The age-adjusted estimate for MDA awareness was 90.8% of those aged ≥5 years (or their parent/guardian), highest in ROU (96.4%), followed by AUA (92.0%), SAV (89.6%), and NWU (87.6%). Of those who were eligible for MDA, an age-adjusted estimate of 89.0% were offered MDA (program reach), and of those who were offered MDA, 99.0% reported taking all the pills (compliance). Age-adjusted coverage was 80.2% of the total population (epidemiological coverage) and 83.9% of the eligible population (program coverage) (Fig 3 and Table 2).

**Table 2:** MDA awareness, reach, compliance and coverage.

	Definition	Participant age group used for assessment ^a^	Survey results ^b^ (‘yes’/total)	Percentage (Unadjusted)	Percentage (Age-adjusted)
**Awareness**	Proportion of total population who knew about MDA	≥5 years	3643/3933	92.6	90.8
**Program reach (of eligible population)**	Proportion of eligible population who were offered MDA	≥5 years	3586/3922	91.4	89.0
**Program reach (of total population)**	Proportion of total population who were offered MDA	≥5 years	3586/3933	91.2	88.6
**Compliance**	Proportion of population offered MDA pills who took all pills	≥5 years	3563/3586	99.4	99.0
**Program coverage (coverage of eligible population)**	Proportion of eligible population who swallowed all MDA pills	≥2 years	3727/4202	88.7	83.9
**Epidemiological coverage (coverage of total population)**	Proportion of total population who swallowed all MDA pills	All ages	3727/4411	84.5	80.2

a. Questions on awareness of MDA and whether offered MDA only asked for participants aged ≥5 years; participants aged 2–4 years only asked coverage questions (Fig 3)

b. Number of participants indicating ‘yes’ in questionnaire/ Number of participants with data available for measure. Data missing for 8 participants.

**Fig 4:**
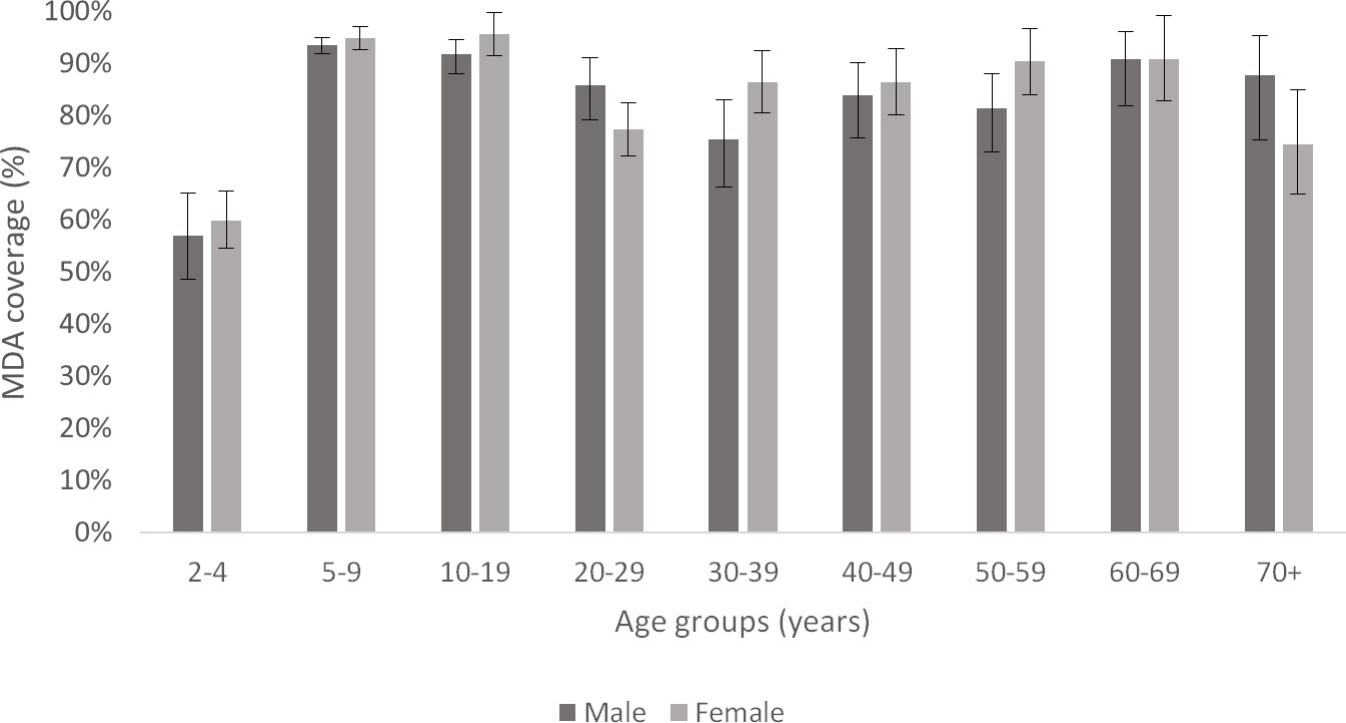
2018 MDA coverage rates (of total population), grouped by age groups and sex. Error bars represent 95% confidence intervals.

MDA coverage was lowest in pre-school children aged 2–4 years (58.6%; 164/280) and highest in those aged 10–19 years (93.8%; 549/585) (Fig 4). There was no significant difference in overall coverage (of total population) between males (79.4%, 95% CI 77.4–81.3%) and females (80.8%, 95% CI 79.1–82.4%). Additionally, in children aged 5–9 years, there was no significant difference in coverage rates between randomly selected households (93.5%; 374/400) and the convenience survey (94.3%; 1454/1542) (p = 0.8).

There was complete concordance in within-household MDA coverage in 56% (280/499) of households (i.e. household members either all did or all did not take MDA), and in a further 19% (95/499) of households there was 80 to <100% concordance. The proportion of participants with indelible ink marks gradually declined from 39.1% to 4.1% as the survey progressed, indicating that the ink was fading over time; we were therefore unable to use the presence of ink marks to validate self-report of MDA participation.

#### Geographical variation in MDA coverage

**Fig 5:**
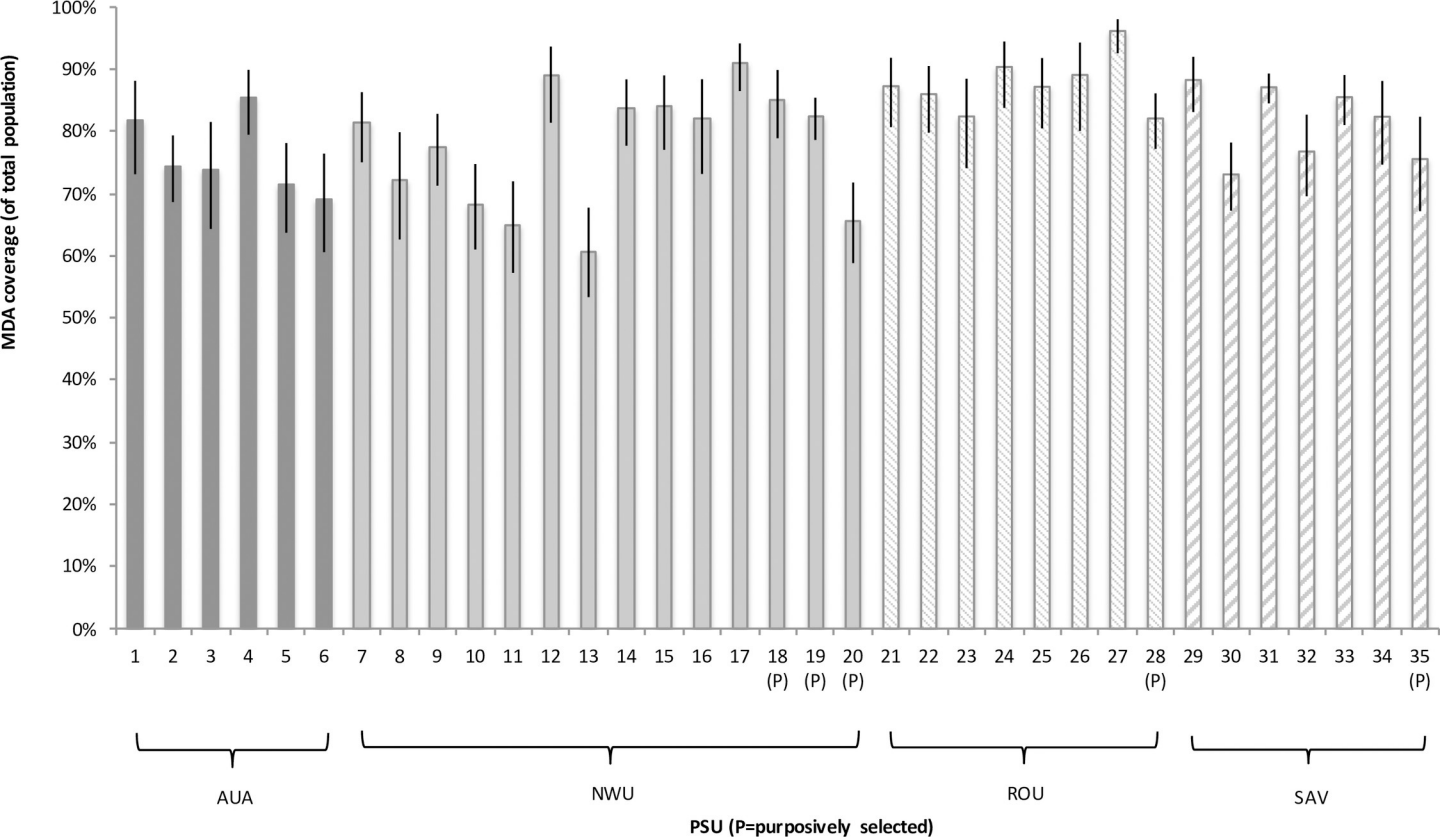
MDA coverage of total population by PSU and Region.

**Fig 6:**
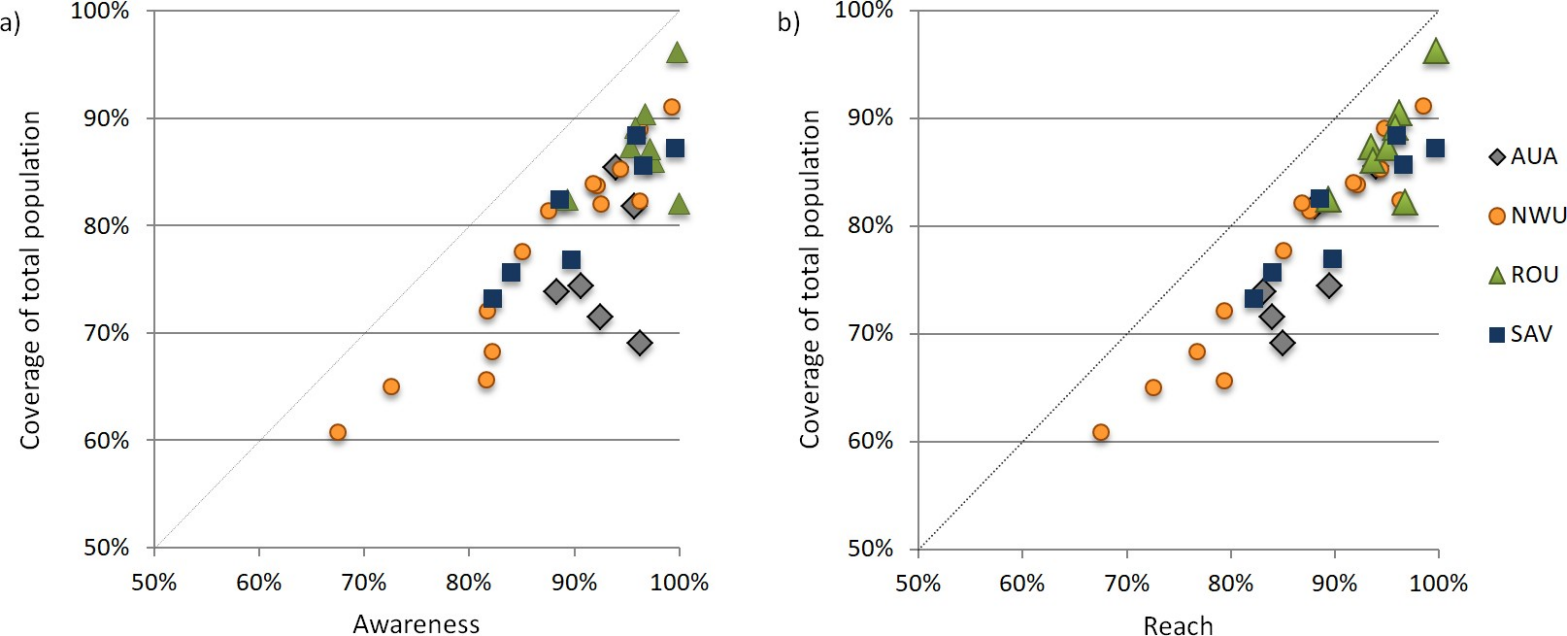
Correlation at each PSU for a) MDA awareness and coverage, and b) MDA reach and coverage.

MDA coverage varied significantly between regions, with the highest age-adjusted coverage rates (of the total population) in ROU (88.1%, 95% CI 85.8–90.1%), followed by SAV (80.3%, 95% CI 77.4–82.9%), NWU (78.1%, 95% CI 75.9–80.2%), and AUA (74.6%, 95% CI 70.1–77.8%). At the regional level, there was no significant association between Ag prevalence and coverage. MDA coverage also varied between PSUs, with age-adjusted rates (of total population) ranging from 60.8% to 92.6% (Fig 5). There was no significant difference in age-adjusted coverage rates between randomly (80.5%, 95% CI 79.1–81.8%) and purposively sampled (77.1%, 95% CI 73.4–80.3%) PSUs. At the PSU level, there was a correlation between awareness and coverage (R^2^ 0.68) and reach and coverage (R^2^ 0.86) (Fig 6). Two PSUs in AUA region and one in ROU stood out as having high awareness but relatively low coverage (Fig 6).

#### Intra-cluster correlation

**Fig 7:**
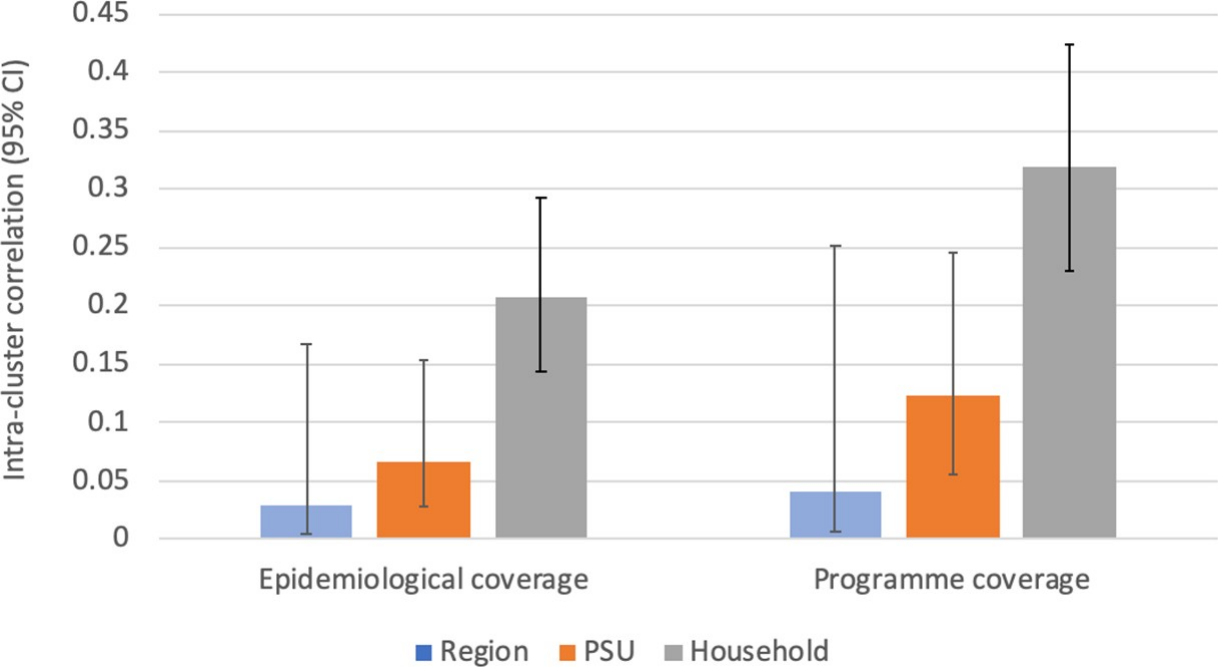
Intra-cluster correlation coefficients for epidemiological coverage and program coverage at region, PSU, and household levels.

After adjusting for age and gender, ICC at the household level was 0.21 (95% CI 0.14–0.29) for epidemiological coverage and 0.32 (95% CI 0.23–0.42) for program coverage. ICC was highest at the household level, followed by PSU and region, suggesting that coverage was more similar between household members compared to those who lived in the same PSU or region. Fig 7 summarizes the ICCs at region, PSU, and household levels for epidemiological coverage and program coverage.

#### Reasons for non-participation and non-compliance

Among eligible participants aged ≥5 years who knew about the MDA (n = 3643), 46 (12.6%) participants reported not being offered MDA because the distributors never came, or because they were away, travelling or working. No-one reported that the MDA supply ran out. Among eligible participants aged ≥5 years who were offered MDA (n = 3586), 23 reported declining the medications. The most common reasons given for not wanting to take the tablets was because they were not sick (n = 5), being worried about side effects (n = 2), didn’t trust the MDA program (n = 2), and didn’t like the taste of pills (n = 1). Two participants who were offered MDA reported not taking any pills, with reasons given being that there were too many pills (n = 1) and no reason given (n = 1). Three participants reported taking only some of the pills, with reasons given being that there were too many pills (n = 1), they had trouble swallowing all the pills (n = 1), and that one pill was lost (n = 1).

#### Participation in previous rounds of MDA

In AUA, ROU and SAV regions, where MDA was last conducted in 2008 and 2011, 86.3% (802/930) of participants aged ≥10 years (i.e. old enough to have previously participated) and who were resident in Samoa at the time, reported participation in MDA in a previous round.

In NWU, where MDA rounds were additionally implemented in 2015 and 2017, among participants who were resident in Samoa at the time, 69.3% (1110/1602) aged ≥5 years and 87.4% (699/800) aged ≥10 years reported participation in previous MDA rounds.

#### Characteristics of people who had never participated in MDA

There were 51 people (2.6% of participants aged ≥10 years) who reported not participating in the 2018 MDA or any previous MDA. Among this cohort, there was equal sex distribution and the highest proportion were in the 10–19 years (33.3%) and 20–29 years (19.6%) age groups. A higher proportion of participants from NWU reported never participating in MDA (3.8%) than from other regions (AUA 0.27%; ROU 1.02%; SAV 1.07%). The most common reason given for not participating in the 2018 MDA was that they didn’t know about it (n = 41, 80%). Overall, Ag prevalence was higher (5.8%) in participants who reported never taking MDA, compared to those who reported taking MDA in 2018 and/or previous rounds (4.9%), but the difference was not statistically significant.

### Self-reported Adverse Events

Of those who participated in MDA and were aged ≥5 years, 83.8% (2986/3563) reported that they did not feel unwell after taking MDA, 13.3% (476/3563) reported feeling unwell but being able to do normal everyday activities (mild AE), and 2.9% (103/3563) reported feeling unwell and being unable to do normal everyday activities, such as going to work or school (moderate or severe AE). Feeling unwell after MDA was more likely to be reported in those aged 20–29 (22%) and 30–39 (25%) years compared to other age groups (p = 0.001), and in females (17.7%) compared to males (15.6%) (p = 0.01). There was no statistically significant difference (p = 0.149) in the reported incidence of AEs between Ag-positive participants (5.6%) and Ag-negative participants (2.8%) who took MDA (Table 3).

**Table 3:** Reported adverse events by antigen positivity.

	Ag positivity			All participants ≥5 years* n (% [95% CI])
	Ag-positive n (% [95% CI])	Ag-negative n (% [95% CI])	Ag unknown n (% [95% CI])	
**Did not feel unwell after taking MDA**	84 (78.5 [69.5–85.9])	2850 (84.2 [82.9–85.4])	52 (73.2 [61.4–83.1])	2986 (83.8 [82.6–85.0])
**Felt unwell after taking MDA**	23 (21.5 [14.1–30.5])	537 (15.9 [14.6–17.1])	19 (26.8 [16.9–38.6])	579 (16.3 {15.1–17.5])
Still able to do normal everyday activities (mild AE)	17 (15.9 [9.5–24.2])	441 (13.0 [11.9–14.2])	18 (25.3 [15.7–37.1])	476 (13.3 [12.2–14.5])
Unable to do normal everyday activities such as going to work or school (moderate or severe AE)	6 (5.6 [2.1–11.8])	96 (2.8 [2.3–3.4])	1 (1.4 [0.0–7.5])	103 (2.9 [2.4–3.5)
**Total**	**107**	**3385**	**71**	**3563**

* Data on adverse events were not collected from participants aged <5 years.

## Discussion

In 2018, Samoa was the first country to distribute nationwide triple drug MDA, and this paper reports important results from a community survey of program awareness, reach, coverage, compliance, and self-reported adverse events. This study demonstrates high reported community awareness, reach, and acceptance of MDA in Samoa, likely to be a result of the significant efforts that were put into community awareness and mobilization, advocacy campaigns, and stakeholder engagement. Overall, support of the program was high and mistrust of the program was extremely low, which indicates high community acceptance and a successful education campaign. In other settings, a fear of side effects or poor understanding of LF infection and transmission has resulted in low compliance [7,21]. Our results demonstrate the importance of both program awareness and reach on coverage, and highlight the importance of community engagement in achieving coverage targets.

In the SaMELFS community survey, which included ~2.2% of the total population, age-adjusted epidemiological coverage was found to be 80.2%, which was well above the recommended target coverage threshold of ≥65% [11]. We found some geographic variation in awareness and coverage, with NWU having the lowest awareness and AUA the lowest coverage rates. Previous nationwide MDAs in Samoa between 2006 and 2011 reported coverage of 80–90% (Samoa MOH data). In 2015 and 2017, MDA was distributed to the NWU region only, with reported coverage of 70–72% (Samoa MOH data). The addition of ivermectin and the larger number of tablets with triple-drug MDA did not seem to affect compliance, with only three people reporting this as a reason for not participating or not taking all of the tablets. Coverage estimated in the current survey was much higher than that previously reported by Joseph et al, where only 48% of 309 children aged 7–10 years reported taking MDA in five villages (biased towards high prevalence villages) in Samoa in 2008 after the main series of two-drug MDAs [22]. However, this surveyed young children in 2008 about MDA participation in 2006 or earlier (when they were less than 8 years old), and results may not be reliable.

Two of the villages surveyed in SaMELFS 2018 were also included in the MOH’s SCT. In Faleasiu, coverage was reported as 95% (114/120) in SaMELFS and 90% (18/20) in the SCT survey. In Leauva’a, coverage was reported as 96.3% (130/135) in SaMELFS and 95% (19/20) in the SCT (S2 Table). The SCT is not designed to provide a reliable estimate of coverage, but both villages were classified as “good coverage” using the SCT, and had high reported coverage rates in the SaMELFS survey.

For community acceptance of MDA, medications must be well-tolerated and side effects must occur at an acceptably low level. AEs have been observed at higher rates in Mf-positive individuals and are expected to occur at higher rates following MDA in communities with high LF prevalence [17,23,24]. Given its higher antifilarial activity, it has been postulated that IDA could be associated with higher rates of AEs than 2-drug treatment using DA [23]. Indeed, clinical trials have demonstrated higher AE rates [24,25], although a recent large multi-center open-label cluster-randomized safety study reported no significant difference in AEs between IDA and DA [23]. In the SaMELFS study, we found a self-reported mild AE rate of 13.3% and a moderate or severe AE rate of 2.9%, but no significant difference in rates of AEs between Ag-positive and Ag-negative persons.

It is difficult to compare our AE findings to existing literature, as rates vary significantly depending on survey method, timing of data collection and population characteristics, with passive data collection yielding lower rates than active collection. In previous randomized trials of IDA, AEs were reported in 59% [25] and 83% [24] of Mf-positive participants, while Weil et al (2019) found a rate of 12% for any AEs in their large multi-center safety study with active follow-up [23]. The study reported an overall prevalence of 1.1% for moderate to severe AEs (Grade 2 and above), with variation between study sites. In Papua New Guinea, 2.4% of participants reported moderate to severe AEs [23], which is similar to the 2.9% (95% CI 2.4–3.5%) reported in the SaMELFS community survey in Samoa. Also, it is important to note that our data from Samoa were collected from direct and targeted questioning about AEs, so prevalence is likely to be higher than studies that relied on passive data collection. Another recent open-label cohort study of 56 participants in Côte d’Ivoire reported AEs in 28% of infected (Ag-positive and Mf-positive) and 25% of uninfected (Ag-negative and Mf-negative) individuals, with all reported AEs being mild [26].

The SaMELFS community survey had several strengths, including sampling a large number of participants of all age groups across Samoa, as well as collecting detailed information on differences in coverage between age groups and regions. The sampling strategy ensured that the participants sampled were broadly representative of population distribution across Samoa, but also that LF ‘hotspots’ were included. Using bilingual local field teams to liaise with community leaders and collect data ensured that the study was culturally appropriate and well accepted by participants, so that recruitment targets were consistently met.

We acknowledge some limitations of our study. The SaMELFS study was a community survey rather than a clinical trial, and was conducted 7–11 weeks after the MDA round. MDA participation was self-reported, and it was not possible to validate self-report by examination of fingernail ink marks as these had largely faded. It is possible that participants did not correctly report MDA participation and/or AEs due to social desirability bias, recall bias, or other reasons. Additionally, children aged 5–9 years sampled via the convenience surveys may have differed from the general population with higher parental awareness of LF or health literacy, leading to a selection bias. However, the absence of statistically significant differences in reported MDA coverage between children aged 5–9 years sampled via the convenience survey and the households, suggests that it is unlikely for any biases to have affected our results. Although households were randomly sampled, it is possible that households unable to be surveyed due to no one being home when the team visited (and thus replaced with an inhabited household), may also have been more likely to miss the MDA due to working away from home, resulting in an overestimate of the coverage rate. Also, those who were ineligible for MDA (e.g. pregnant, unwell) might have been less likely to agree to participate in the survey, and therefore not included in our estimates of awareness, reach, compliance, and coverage. Our study assessed self-reported AEs 7–11 weeks after MDA and it was not possible to reliably differentiate between moderate and severe AEs. Additionally, most of our field team were not clinically trained, so we did not attempt to assess duration, causality, or severity beyond whether participants were still able to do everyday activities. Thus, results may be subject to recall bias (exacerbated by the time delay), or incorrect attribution of unrelated symptoms to the MDA. Antigenaemia was measured 7–11 weeks after MDA, but Ag persists for at least months after treatment and our results should represent prevalence prior to the MDA [24–26]. A limitation of coverage surveys in general is that results for children are provided by parents or guardians. While we report coverage for children as young as two years, our results for young children were of awareness in their parents/guardians. Also, parents may not be have been entirely sure about whether children took MDA at school. In the household survey, care was taken to obtain specific answers from each individual, but it was possible that answers might have been influenced by the presence of other household members and their answers.

The success of the 2018 MDA delivery in Samoa was due to a collaborative effort among stakeholders, successful community engagement and mobilization, and a multi-location delivery strategy. The experience in Samoa demonstrates the feasibility and safety of countrywide IDA for the elimination of LF.

## Conclusion

This cross-sectional community survey of triple-drug MDA coverage and self-reported adverse events following the 2018 MDA round in Samoa demonstrated a high reported program awareness, reach, coverage, and compliance, and found that the MDA was well accepted and tolerated. Given the need for renewed efforts to eliminate LF using IDA to accelerate GPELF’s progress toward 2030 programmatic goals, our results are encouraging for Samoa’s ongoing MDA program and for other countries that are also planning to implement IDA.

## Supporting information

S1 STROBE checklist (DOCX)

S1 Fig Study participant flowchart, as shown in Fig 3 of main text, with each component labelled alphabetically. (TIF)

S1 Table Weight-based dosing schedule for triple-drug MDA in Samoa, 2018. (DOCX)

S2 Table Results of Supervisor’s Coverage Tool (SCT) in three selected villages. (DOCX)

S3 Table Age and sex distribution of persons presenting with an adverse event to a public health facility from Samoa Ministry of Health surveillance. (DOCX)

S4 Table Presenting symptoms of persons with an adverse event attending a public health facility from Samoa Ministry of Health surveillance. (DOCX)

S5 Table Formulas for calculating MDA awareness, reach, compliance and coverage from different study participants using notation from flowchart in S1 Fig. (DOCX)
