# Supplementary material for: A community survey of coverage and adverse events following country-wide triple-drug mass drug administration for lymphatic filariasis elimination, Samoa 2018
Source: PLoS Negl Trop Dis. 2020 Nov 30;14(11):e0008854. doi: 10.1371/journal.pntd.0008854 (PMC7728255; doi:10.1371/journal.pntd.0008854)
Supplement: S1 Table — (DOCX) [file pntd.0008854.s003.docx]

S1 Table. Weight-based dosing schedule for triple-drug MDA in Samoa, 2018.

| **Weight range (kg)** | **Number of ivermectin tablets (3mg)** | **Number of DEC tablets (100mg)** | **Number of albendazole tablets (400mg)** | **Total number of tablets** |
| --- | --- | --- | --- | --- |
| <15 kg (or 2-4 years old) | 0 | 1 | 1 | 2 |
| 15-23 kg | 1 | 1 | 1 | 3 |
| 24-38 kg | 2 | 2 | 1 | 5 |
| 39-53 kg | 3 | 3 | 1 | 7 |
| 54-68 kg | 4 | 4 | 1 | 9 |
| 69-83 kg | 5 | 5 | 1 | 11 |
| 84-98 kg | 6 | 6 | 1 | 13 |
| 99-124 kg | 7 | 7 | 1 | 15 |
| >124kg | 8 | 8 | 1 | 17 |
