# Supplementary material for: A community survey of coverage and adverse events following country-wide triple-drug mass drug administration for lymphatic filariasis elimination, Samoa 2018
Source: PLoS Negl Trop Dis. 2020 Nov 30;14(11):e0008854. doi: 10.1371/journal.pntd.0008854 (PMC7728255; doi:10.1371/journal.pntd.0008854)
Supplement: S2 Table — (DOCX) [file pntd.0008854.s004.docx]

S2 Table. Results of Supervisor’s Coverage Tool (SCT) in three selected villages.

| **Village** | **Age range** | **Male**  n (%) | **Offered MDA drugs**  n (%)^a^ | **Swallowed MDA drugs**  n (%) | **Where MDA drugs accessed** | **Finger mark present (% of those who reported taking MDA)^b^** |
| --- | --- | --- | --- | --- | --- | --- |
| Faleasiu | 2-70 | 10 (50) | 18 (90) | 18 (90) | 14 Home;  2 School;  2 Other village | 12 (66.7) |
| Leauva’a | 3-74 | 12 (60) | 19 (95) | 19 (95) | 8 Home;  7 School;  1 Fixed health facility;  3 Other | 19 (100) |
| Nofoali’i | 3-81 | 11 (55) | 20 (100) | 20 (100) | 14 Home;  3 School;  1 Church;  2 Other | 17 (85) |
| **All villages** | **2-81** | **33 (55)** | **57 (95)** | **57 (95)** | **36 Home;  12 School;  9 Other** | **48 (84)** |

1. Reasons for the three people who reported not being offered MDA and not taking MDA were being away at the time (n=1), being sick (n=1), and parents not being home (n=1).
2. Reasons given for the absence of ink marks included “scratched it”, “faded”, and “team forgot”.
