# Supplementary material for: A community survey of coverage and adverse events following country-wide triple-drug mass drug administration for lymphatic filariasis elimination, Samoa 2018
Source: PLoS Negl Trop Dis. 2020 Nov 30;14(11):e0008854. doi: 10.1371/journal.pntd.0008854 (PMC7728255; doi:10.1371/journal.pntd.0008854)
Supplement: S3 Table — (DOCX) [file pntd.0008854.s005.docx]

S3 Table. Age and sex distribution of persons presenting with an adverse events to a public health facility from Samoa Ministry of Health surveillance.

| **Age group (years)** | **Male** | **Female** | **Total** |
| --- | --- | --- | --- |
| 2-10 | 16 | 12 | 28 |
| 11-20 | 8 | 1 | 9 |
| 21-30 | 2 | 2 | 4 |
| 31-40 | 3 | 5 | 8 |
| 41-50 | 2 | 3 | 5 |
| 51-60 | 2 | 1 | 3 |
| 61+ | 5 | 3 | 8 |
| **Total** | **38** | **27** | **65** |
