# Supplementary material for: A community survey of coverage and adverse events following country-wide triple-drug mass drug administration for lymphatic filariasis elimination, Samoa 2018
Source: PLoS Negl Trop Dis. 2020 Nov 30;14(11):e0008854. doi: 10.1371/journal.pntd.0008854 (PMC7728255; doi:10.1371/journal.pntd.0008854)
Supplement: S4 Table — (DOCX) [file pntd.0008854.s006.docx]

S4 Table. Presenting symptoms of persons with an adverse events attending a public health facility from Samoa Ministry of Health surveillance.

| **Reported symptom (s)** | **Number** | **%** |
| --- | --- | --- |
| Dizziness, lethargy, and nausea | 12 | 18 |
| Dizziness and nausea | 10 | 15 |
| Generalised itchy body rash | 10 | 15 |
| Dizziness, lethargy, nausea and vomiting | 8 | 12 |
| Non-itchy rash | 8 | 12 |
| Malaise and vomiting | 6 | 9 |
| Generalised body pains | 6 | 9 |
| Death during MDA but deemed unrelated to MDA | 4 | 5 |
| Abdominal pain | 1 | 2 |
| Chest pain, shortness of breath, and headache | 1 | 2 |
| **Total** | **65** | **100** |
