# Supplementary material for: A community survey of coverage and adverse events following country-wide triple-drug mass drug administration for lymphatic filariasis elimination, Samoa 2018
Source: PLoS Negl Trop Dis. 2020 Nov 30;14(11):e0008854. doi: 10.1371/journal.pntd.0008854 (PMC7728255; doi:10.1371/journal.pntd.0008854)
Supplement: S5 Table — (DOCX) [file pntd.0008854.s007.docx]

S5 Table. Formulas for calculating MDA awareness, reach, compliance and coverage from different study participants using notation from flowchart in S1 Fig.

|  | | **Definition** | **Participant age group used for assessment ^a^** | **Survey results ^b^**  **(‘yes’/total)** | **Formula** |
| --- | --- | --- | --- | --- | --- |
| **Awareness** | | Proportion of total population who knew about MDA | ≥5 years | 3643/3933 | f / (d-h) |
|  | |  |  |  |  |
| **Program reach  (of eligible population)** | | Proportion of eligible population who were offered MDA | ≥5 years | 3586/3922 | l / (d-h-n) |
|  | |  |  |  |  |
| **Program reach  (of total population)** | | Proportion of total population who were offered MDA | ≥5 years | 3586/3933 | l / (d-h) |
|  |  |  |  |  |  |
| **Compliance** | | Proportion of population offered MDA pills who took all pills | ≥5 years | 3563/3586 | o / l |
|  | |  |  |  |  |
| **Program coverage  (coverage of eligible population)** | | Proportion of eligible population who swallowed all MDA pills | ≥2 years | 3727/4202 | (i+o) / (c–h-k-n) |
|  | |  |  |  |  |
| **Epidemiological coverage  (coverage of total population)** | | Proportion of total population who swallowed all MDA pills | All ages | 3727/4411 | (i+o) / (a-h-k) |
